# Risk of renal events following intravenous iodinated contrast material administration among inpatients admitted with cancer a retrospective hospital claims analysis

**DOI:** 10.1186/s40644-018-0159-3

**Published:** 2018-08-24

**Authors:** Chaan S. Ng, Sanjeeva P. Kalva, Candace Gunnarsson, Michael P. Ryan, Erin R. Baker, Ravindra L. Mehta

**Affiliations:** 1 MD Anderson Cancer Center, 1515 Holcombe Boulevard, Houston, TX 77030-4009 USA; 2 University of Texas Southwestern Medical Center, 5323 Harry Hines Blvd, Dallas, 75390-8834 TX USA; 3 CTI Clinical Trial & Consulting Services100 E, RiverCenter Blvd, Covington, KY 41011 USA; 4 University of California San Diego 0892 UCSD Medical Center, 9500 Gilman Drive, La Jolla, CA 92037 USA

**Keywords:** Iodinated contrast media, Acute renal event, Cancer, Contrast-induced nephropathy, Contrast-induced acute kidney injury, Computed tomography

## Abstract

**Background:**

There is little published evidence examining the use of contrast material (CM) and the risk of acute renal adverse events (AEs) in individuals with increasingly common risk factors including cancer and chronic kidney disease (CKD). The objective of this study was to use real world hospital data to test the hypothesis that inpatients with cancer having CT procedures with iodinated CM would have higher rates of acute renal AEs in comparison to inpatients without cancer.

**Methods:**

Inpatient hospital visits in the Premier Hospital Database from January 1, 2010 through September 30, 2015 were eligible for inclusion. The outcome of interest was a composite of acute renal AEs including: acute kidney injury, acute renal failure requiring dialysis, contrast induced-acute kidney injury and renal failure. Multivariable models, adjusted for differences in patient demographics and comorbid conditions, were used to estimate the incremental risk of acute renal AEs by CT (with or without iodinated CM), CKD stage and type of cancer.

**Results:**

Among 29,850,475 inpatient visits across 611 hospitals, 7.4% had record of a CT scan, 5.9% had CKD, and 3.4% had the primary diagnosis of cancer. The baseline risk for an acute renal AE in patients without cancer or CKD and no CT or CM was 0.5%. The absolute risk increases from baseline by 0.2% with a CT and by 0.8% with iodinated CM. Patients with CKD having a CT scan with iodinated CM have an absolute risk of 4.1 to 9.7% depending on the stage of CKD. For patients with cancer, the absolute risk increases, varying from 0.3 to 2.3% depending on the type of cancer.

**Conclusions:**

Inpatients with cancer are at higher likelihood of developing acute renal AEs following CT with iodinated CM compared to those without a cancer. Understanding the underlying risks of acute renal AEs among complex inpatient admissions is an important consideration in treatment choices for oncology patients.

## Background

Adverse events (AEs) following intravascular administration of iodinated contrast material (CM) occur in 0.02 to 0.04% of patients. These include kidney injury, respiratory or cardiac arrest, convulsions, and loss of consciousness [1–3]. Renal insufficiency has been noted as both contributing to the risk of a post-CM AEs and as a result thereof [4–6]. However, the incidence of nephropathy specifically caused by iodinated CM is not well understood. As noted by the American College of Radiology, most published studies focus on the diagnosis of post-contrast acute kidney injury (PC-AKI), which is defined as sudden deterioration in renal function within 48 h following the intravascular administration of iodinated CM. PC-AKI is a correlative diagnosis, a subset of PC-AKI cases are contrast-induced nephropathy (CIN or CI-AKI), which is a causative diagnosis [7]. CI-AKI is commonly defined as an increase in serum creatinine (SCr) greater than 25% or 44.2umol/L (0.5 mg/dL) from baseline within 2 or 3 days of intravascular CM administration in the absence of an alternative cause [5, 8]. CI-AKI has an estimated incidence of 8 to 20% of cancer patients who undergo contrast-enhanced CT [6, 9–11]. However, most studies do not include a control group for analysis, which is problematic due to the variation in SCr observed in hospitalized patients regardless of CM administration [5]. Depending on the definition utilized, AKI has been reported in 6 to 35% of inpatients without CM exposure [5, 12].

Cancer treatments as well as the timing of treatment and CT imaging have been investigated as risk factors for acute reactions to iodinated CM [13, 14]. Other than chronic kidney disease (CKD), risk factors for CI-AKI include diabetes, hypertension, malignancy, age > 65 years, use of non-steroidal anti-inflammatory drugs, and timing of CT within 45 days after last chemotherapy [9, 15]. Regardless of the cause, cancer patients who develop renal failure may have worse prognosis and survival [16–19].

While the biomedical literature indicates that the rate of AEs associated CM use is low, there is little evidence examining the use of CM and the risk of renal AEs in individuals with increasingly common risk factors including cancer and CKD. The objective of this study was to use real world hospital data to test the hypothesis that patients with cancer having CT with iodinated CM would have higher rates of acute renal AEs than those without cancer.

## Methods

### Data source

Data for the study were derived from the Premier Hospital Database, which currently contains data from more than 350 million patient encounters, or one in every five discharges in the United States (US) [20]. The database contains data from standard hospital discharge files, including a patient’s demographic and disease state, and information on billed services, including medications, laboratory, diagnostics and therapeutic services in de-identified patient daily service records. In addition, information on hospital characteristics, including geographic location, bed size and teaching status are also available. Preliminary comparisons between patient and hospital characteristics for the hospitals included in the database and those of the probability sample of hospitals and patients selected for the National Hospital Discharge Survey (NHDS) suggest that the patient populations are similar with regard to patient age, gender, length of stay, mortality, primary discharge diagnosis, and primary procedure groups [21]. All data used to perform this analysis were de-identified and accessed in compliance with the Health Insurance Portability and Accountability Act. As a retrospective analysis of a de-identified database, the research was exempt from IRB review under 45 CFR 46.101(b)(4).

### Inclusion/exclusion criteria

Any inpatient hospital visit in the Premier Hospital Database from January 1, 2010 through September 30, 2015 was eligible for inclusion. Inpatient was defined as a visit which included an overnight stay. Patient visits were excluded if a patient had a record of end stage renal disease requiring dialysis (ESRD ICD-9 code: 585.6), kidney transplantation (ICD-9 code: V42.0, 996.81, or 55.6×) or AKI (ICD-9 code: 584.9) upon admission (determined by a variable that indicated the patient had the condition upon admission). To isolate the risk of renal events among oncology patients hospitalized for diagnosis or treatment of cancer, visits with a secondary or historical diagnosis of cancer were excluded. Visits where the primary diagnosis or reason for the inpatient stay was cancer were included (Table 5 in Appendix).

### Variables of interest

Patient visits with a record of primary cancer were further categorized by the following types of cancer: Bone, Breast, Colorectal, Endocrine, Gastrointestinal, Gynecological, Hemolymph, Leukemia, Liver, Lung, Neurological, Respiratory, Skin, Urinary and Miscellaneous (rare cancers).

The primary outcome of interest was a composite of adverse renal events, defined as one or more of the following: AKI, acute renal failure requiring dialysis, CI-AKI or renal failure (ICD-9 codes Table 6 in Appendix). Acute renal events were identified as being outcomes if there was a record of the event during the hospitalization and the event of interest was not recorded as present on admission.

To identify usage of CM, keyword text mining was performed on patients’ charge master billing files. Using product brand names and generic keywords for CM use, the following categories were created: iodinated, non-iodinated, or unknown type. If no evidence of CM usage was found on the visit, the visit was assumed to have no CM usage. CM usage could have occured during a CT or CTA scan, see Table 7 of Appendix for codes used to define CT and CTA scans.

In order to quantify the effect of CKD, a dichotomous variable was made for CKD status based on the presence of CKD stage recorded in the visit. Additionally, an ordinal variable was created for CKD stage (0 = no disease, stage 1, stage 2, stage 3, stages 4 &5) (Table 8 in Appendix). It is important to note, that patients with unspecified CKD were only included in the dichotomous variable and excluded in the staging variable due to the non-specificity of their renal disease status.

The following variables were summarized prior to statistical modeling: patient demographics (age, race, gender, insurance type, and admission type), visit characteristics (whether or not the patient underwent a CT, CM usage and type), patient conditions (primary cancer, type of cancer, CKD severity, and overall disease severity as measured by the Elixhauser Comorbidity Index (ECI Table 9 in Appendix)) [22]. All components of the composite of renal AEs were described prior to multivariable modeling by the following key model inputs: CKD by severity, CT (with or without iodinated CM) and cancer type.

### Statistical analyses

All multivariable renal AE models adjusted for differences in both patient demographics and comorbid conditions. The hospital fixed-effects specification was used to account for time-invariant variation across a hospital that was otherwise unobservable. This methodological choice was made to compensate for the non-random relationship between patients and hospital choice which may result in variation across hospitals in both patient mix (e.g. the share and severity of oncology patients) and in the rate of renal events which may lead to a spurious correlation. By limiting the analysis to variation within hospitals, we study patients treated in a similar environment using similar standards of care and hospital protocols. The decision to utilize a particular product or drug during a hospital visit may depend on formal hospital guidelines, physician practice patterns or preferences, negotiated reimbursement schedules with insurance companies, and other local (geographic and/or hospital) characteristics.

All analysis was performed in SAS version 9.4 (Cary, NC).

## Results

**Fig. 1:**
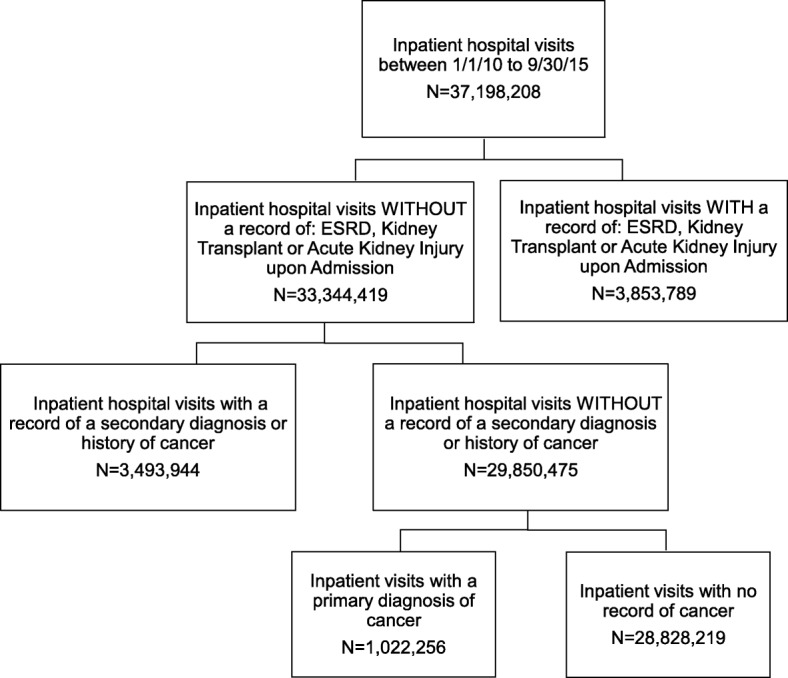
Attrition Diagram

A total of 29,850,475 inpatient visits across 611 hospitals met the study inclusion criteria (Fig. 1). The average age of patients at the time of the inpatient visit was 45 years (standard deviation (sd) 27.5). The majority of patients were female (60%), Caucasian (65%), and the most frequent insurer was Medicare (34%). Emergency and urgent hospitalizations made up 61% of all visits. Overall, 7% of inpatient visits had a record of a CT and 80% of visits had no record of CM (Table 1).

**Table 1:** Patient Visit Characteristics

	Total	
	N	Percent
Total Visits	29,850,475	100%
Age		
Median	48	
Mean	45.0	
Standard deviation	27.50	
Race		
Caucasian	19,314,454	64.7%
African-American	4,052,601	13.6%
Other	6,483,420	21.7%
Gender		
Female	17,831,769	59.7%
Male	12,015,263	40.3%
Unknown	3443	0.0%
Insurance		
Commercial	1,681,308	5.6%
Medicare	10,010,108	33.5%
Medicaid	6,968,569	23.3%
Managed Care	8,043,140	26.9%
Other	3,147,350	10.5%
Admission Type		
Emergency	13,780,883	46.2%
Urgent	4,466,926	15.0%
Elective	7,507,444	25.2%
Other/Unknown	4,095,222	13.7%
CT Scan	2,195,374	7.4%
Contrast Used		
Iodinated	2,290,183	7.7%
Non-Iodinated	463,956	1.6%
Both	73,839	0.2%
Unknown	3,258,046	10.9%
None	23,764,451	79.6%

The population had a mean ECI score of 2.1 (sd 2.17), comorbid conditions and frequencies are shown in Table 2. Among the 6% of visits with CKD, the CKD stage was: stage 1 (0.7%), stage 2 (5.6%), stage 3 (36.5%), stage 4/5 (12.4%) and stage unspecified (44.8%). Cancer was the primary diagnosis in 3.4% of visits. The highest percentage of primary cancer visits reported were: gastrointestinal (16.1%), urinary (14.6%) and lung (13.1%).

**Table 2:** Patient Comorbidities

	Total		
	N	Percent	
Total Visits	29,850,475	100%	
Elixhauser Comorbidities			
Congestive Heart Failure	2,956,976	9.9%	
Cardiac Arrhythmia	4,708,604	15.8%	
Valvular Disease	1,269,470	4.3%	
Pulmonary Circulation Disorders	896,999	3.0%	
Peripheral Vascular Disorders	1,392,847	4.7%	
Hypertension (Uncomplicated)	10,030,305	33.6%	
Hypertension (Complicated)	1,768,162	5.9%	
Paralysis	470,505	1.6%	
Other Neurological Disorders	2,076,621	7.0%	
Chronic Pulmonary Disease	5,651,859	18.9%	
Diabetes (Uncomplicated)	4,479,120	15.0%	
Diabetes (Complicated)	962,632	3.2%	
Hypothyroidism	2,680,999	9.0%	
Renal Failure	1,781,578	6.0%	
Liver Disease	1,017,975	3.4%	
Peptic Ulcer Disease (excluding bleeding)	219,464	0.7%	
AIDS/HIV	77,709	0.3%	
Lymphoma	50,977	0.2%	
Metastatic Cancer	375,880	1.3%	
Solid Tumor without Metastasis	816,723	2.7%	
Rheumatoid Arthritis Collagen	582,016	1.9%	
Coagulopathy	988,278	3.3%	
Obesity	3,335,095	11.2%	
Weight Loss	985,799	3.3%	
Fluid and Electrolyte Disorders	5,086,695	17.0%	
Blood Loss Anemia	260,342	0.9%	
Deficiency Anemia	711,987	2.4%	
Alcohol Abuse	1,672,862	5.6%	
Drug Abuse	1,684,008	5.6%	
Psychoses	920,047	3.1%	
Depression	4,026,007	13.5%	
Elixhauser Comorbidity Index			
Median	2		
Mean	2.1		
Std Dev	2.17		
Chronic Kidney Disease			
No CKD	28,085,084	94.0%	
CKD	1,765,391	5.9%	
Stage of Chronic Kidney Disease	N	% Overall	% of CKD
Stage 1	11,958	0.0%	0.7%
Stage 2	99,004	0.3%	5.6%
Stage 3	644,398	2.2%	36.5%
Stage 4 & 5	219,255	0.7%	12.4%
Unspecified	790,776	2.6%	44.8%
Diagnosis of Cancer			
No Cancer	28,828,219	97.0%	
Primary Cancer	1,022,256	3.4%	
Type of Primary Cancer	N	% Overall	% of Cancer
Bone	2991	0.0%	0.3%
Breast	77,428	0.3%	7.6%
Colorectal	127,275	0.4%	12.5%
Endocrine	37,769	0.1%	3.7%
Gastrointestinal	164,323	0.6%	16.1%
Gynecological	64,034	0.2%	6.3%
Hemolymph	42,572	0.1%	4.2%
Leukemia	37,869	0.1%	3.7%
Liver	18,022	0.1%	1.8%
Lung	133,837	0.4%	13.1%
Miscellaneous	120,556	0.4%	11.8%
Neurological	29,724	0.1%	2.9%
Respiratory	9034	0.0%	0.9%
Skin	7073	0.0%	0.7%
Urinary	149,749	0.5%	14.6%

*CKD* Chronic Kidney Disease

The unadjusted rates of the renal AE outcome and its components are reported in Table 3 by the following key variables: CKD stage, CT (with or without iodinated CM) and cancer type. The unadjusted baseline rate of the renal AEs was 0.5% for inpatient visits without cancer, CKD or CT and CM. The frequency of renal events increased with CKD severity (0.9% for patients with no record of CKD; 6.1% for a patient with CKD stage 1 to 12.7% among CKD patients stage 4 & 5). Among visits with primary cancer, the unadjusted rate of renal events was 3.0%, an increase from 1.4% in visits with no cancer diagnosis. The unadjusted rate of renal events varied by cancer type: leukemia (5.3%), liver (4.3%), urinary (4.1%), and colorectal (4.1%). When considering all AEs which make up the renal AE composite, AKI without dialysis contributed to the composite more than other components.

**Table 3:** Renal Adverse Events: Prior to Multivariable Modeling (Unadjusted)

	Renal Adverse Event Outcome	Components of the Renal Adverse Events Outcome			
		Acute Kidney Injury without dialysis	Acute Kidney Injury with dialysis	CI-AKI	Renal Failure
Baseline	0.5%	0.5%	0.0%	0.0%	0.0%
No CKD	0.9%	0.8%	0.0%	0.0%	0.0%
CKD Stage 1	6.1%	6.0%	0.1%	0.2%	0.0%
CKD Stage 2	8.4%	8.3%	0.1%	0.2%	0.0%
CKD Stage 3	11.4%	11.1%	0.3%	0.3%	0.1%
CKD Stage 4&5	12.7%	11.6%	0.8%	0.3%	0.2%
CT	2.8%	2.6%	0.1%	0.1%	0.0%
No CT	1.3%	1.3%	0.0%	0.0%	0.0%
CT with Iodinated Contrast	2.9%	2.8%	0.1%	0.1%	0.0%
CT without Iodinated Contrast	2.7%	2.6%	0.1%	0.1%	0.0%
No-Cancer	1.4%	1.3%	0.0%	0.0%	0.0%
Cancer	3.0%	2.9%	0.1%	0.0%	0.0%
Bone	1.4%	1.3%	0.1%	0.0%	0.0%
Breast	0.6%	0.6%	0.0%	0.0%	0.0%
Colorectal	4.1%	4.0%	0.1%	0.0%	0.0%
Endocrine	1.5%	1.5%	0.1%	0.0%	0.0%
Gastrointestinal	3.3%	3.2%	0.1%	0.1%	0.0%
Gynecological	2.5%	2.4%	0.1%	0.0%	0.0%
Hemolymph	3.9%	3.5%	0.3%	0.1%	0.1%
Leukemia	5.3%	4.9%	0.3%	0.1%	0.1%
Liver	4.3%	4.0%	0.2%	0.1%	0.1%
Lung	2.8%	2.7%	0.1%	0.1%	0.0%
Miscellaneous	1.9%	1.9%	0.0%	0.0%	0.0%
Neurological	1.0%	0.9%	0.0%	0.0%	0.0%
Respiratory	2.2%	2.2%	0.0%	0.0%	0.0%
Skin	1.6%	1.6%	0.0%	0.0%	0.0%
Urinary	4.1%	4.0%	0.1%	0.0%	0.0%

*AKI* Acute Kidney Injury, *CKD* Chronic Kidney Disease, *CI-AKI* Contrast induced acute kidney injury

**Fig. 2:**
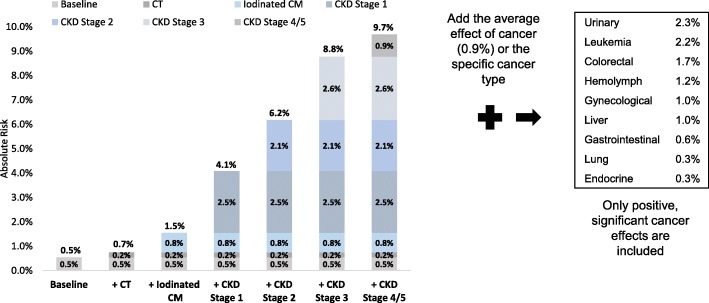
Renal Event: Multivariable Risk Decomposition – CT, CM, CKD, and Cancer Type, The percentages shown in Table 4, and in this figure in the columns and at the top of each column differ slightly due to rounding. +CT = record of CT; +Iodinated = record of iodinated contrast material (CM); +CKD = chronic kidney disease, The absolute risk of a renal event can be calculated based on a patient’s comorbidities. For example, a patient hospitalized for cancer who had a CT scan with iodinated CM and CKD stage 1, had a 4.9% risk of a renal event. The risk was calculated as follows: (baseline [.5%] + CT [.2%] with iodinated CM [.8%] + CKD stage 1 [2.5%] + cancer [.9%])

The fixed effects multivariable models controlled for differences in patient demographics and comorbid conditions and decomposed the risk by the following variables: CT, iodinated CM, CKD stage and cancer type (Table 4 and Fig. 2). Estimates of absolute risk of the renal AEs are reported with confidence intervals for CT, iodinated CM, CKD stage and cancer (Table 4). Absolute risk of an acute renal event increased with non-contrast CT by 0.2%, iodinated CM increased the risk by an additional 0.8%. The increased risk varied by cancer type, overall, the risk of a renal event increased by 0.9%. The risk by individual cancer types range from 0.3% for endocrine cancer to 2.3% for urinary cancers. Absolute risk increased with CKD severity: stage 1 (2.5%), stage 2 (4.6%), stage 3 (7.2%), stage 4 & 5 (8.1%).

**Table 4:** Multivariable Estimates of Absolute risk of an Acute Renal Adverse Event

Variable	Absolute Risk Estimate (95% confidence interval)	*P*-Value
CT	0.19% (0.17, 0.21%)	< 0.0001
Iodinated CM	0.81% (0.80, 0.83%)	< 0.0001
CKD Stage 1	2.55% (2.35, 2.74%)	< 0.0001
CKD Stage 2	4.64% (4.56, 4.71%)	< 0.0001
CKD Stage 3	7.24% (7.19, 7.28%)	< 0.0001
CKD Stage 4/5	8.14% (8.08, 8.19%)	< 0.0001
Cancer	0.87% (0.85, 0.89%)	< 0.0001
Urinary	2.33% (2.28, 2.39%)	< 0.0001
Leukemia	2.20% (2.09, 2.31%)	< 0.0001
Colorectal	1.69% (1.63, 1.75%)	< 0.0001
Hemolymph	1.22% (1.12, 1.33%)	< 0.0001
Gynecological	1.03% (0.95, 1.11%)	< 0.0001
Liver	1.00% (0.84, 1.16%)	< 0.0001
Gastrointestinal	0.59% (0.54, 0.65%)	< 0.0001
Lung	0.33% (0.27, 0.39%)	< 0.0001
Endocrine	0.29% (0.18, 0.39%)	< 0.0001

*CKD* Chronic Kidney Disease, *CM* Contrast material

Figure 2 provides a cumulative visual for the regression estimates reported in Table 4. The first bar in the figure is the absolute risk of the renal AEs at baseline, 0.5%. Baseline risk represents patient visits without CT, CM, CKD or cancer. From left to right, the absolute risk associated with each variable is reported as well as how the risk accumulates with each additional variable. For example, a patient hospitalized for cancer that had a CT scan with iodinated CM and CKD stage 1, had a 4.9% risk of a renal event. The absolute risk of a renal event increases substantially for patients with CKD. Inpatients who underwent a CT with iodinated CM who do not have cancer had the following risk based on CKD severity: stage 1 (4.1%), stage 2 (6.2%), stage 3 (8.8%), stage 4 & 5 (9.7%).

## Discussion

After controlling for patient demographics, comorbid conditions and hospital fixed effects, the risk of an acute renal event for hospitalized patients ranges from 0.5% at baseline (patient visits without CT, CM use, CKD or cancer experiencing AKI) to as high as 10.6% (patient visits with a CT with iodinated CM with CKD stage 4 or 5 and cancer). The increasing risk with CKD stage reflects the previously reported impact of compromised renal function and adds to the literature by showing the risk of renal AEs by cancer type. The effect of a cancer diagnosis on the risk of renal AEs was 0.9%, with specific cancers having up to 2.3% (for urinary cancer) added risk. The incremental risk of a renal event associated with a CT without contrast was 0.2%, which clinically may be counterintuitive. This incremental risk was most likely due to the CT being a proxy for sicker patients or other procedures not controlled for in the regression analysis. Regardless of the reason, the effect is small compared to the other factors.

Large retrospective single center studies have previously explored the risk of intravenous CM via propensity-matched cohort analyses [23, 24]. Such investigations differ from our current analysis in heterogeneity (or homogeneity) of population examined, this study specifically surveyed the inpatient setting while considering the impact of CKD stage and cancer diagnosis.

It is not difficult to surmise why cancer patients may be particularly susceptible to renal events given their high prevalence of renal insufficiency, concomitant nephrotoxic chemotherapeutic regimens, and predisposition to dehydration secondary to advanced age, poor appetite, nausea, and vomiting [25]. It has additionally been suggested that patients with active cancer undergoing CM enhanced CT are particularly at risk of CI-AKI even in the absence of significant renal impairment as underlying renal insufficiency may be masked due to falsely low creatinine concentration resulting from diminished muscle mass [10].

This study did not explore the potential additive effects of different types of CM and chemotherapy; however, it has been suggested that CI-AKI may develop 4.5 times more frequently in cancer patients who undergo recent chemotherapy [9] and that exposure to CM within a week prior to nephrotoxic chemotherapeutic agents, for example cisplatin, significantly increases the risk of nephropathy [26]. Similar nephrotoxic effects of iodinated CM and chemotherapeutic agents upon the renal vasculature may rationalize the amplified risk. Not surprisingly, chemotherapy has been increasingly identified as an additional risk factor, evident by inclusion into CI-AKI consensus statements and guideline recommendations [27].

While the current analysis did not assess renal AEs by class of CM, a recent prospective, multicenter, randomized controlled trial suggested more favorable safety profile of iso-osmolar CM (iodixanol) versus low-osmolar CM (iopromide) in low risk cancer patients defined by eGFR> 60 mL/min [28]. Adequately sized and designed studies of prospective nature are warranted to elucidate findings further.

Our findings quantify absolute risk of renal events and are noteworthy given the marked consequences that AKI may elicit within the oncology setting. Salahudeen et al. recently conducted cross-sectional analysis of prospectively collected data on 3558 patients admitted to the University of Texas, M.D. Anderson Cancer Center and found higher rates of AKI versus most non-cancer settings. In patients with AKI, length of stay (100%), cost (106%), and odds for mortality (4.7-fold) were significantly greater [29].

On account of these implications and due to the complex bidirectional relationship between cancer and kidney function, there is need for further investigation and periprocedural recommendations. The intra-arterial administration of CM within interventional cardiovascular procedures has been investigated at length, with subsequent guideline development central to patient risk assessment, hydration strategies, and emphasis on limiting volumes of CM administered. While it has been suggested that overall risk is lower with intravenous administration of CM, susceptible oncologic settings and vulnerable patients should be identified (particularly a patient’s state of kidney health and timing of treatment or imaging) and integrated strategies should be employed to minimize the risk of renal events among inpatient cancer patients undergoing CT with CM.

The intricate association and increasing prevalence of cancer and AKI/CKD has led to mounting interest in this complex environment and prompted evolution of the novel onco-nephrology subspecialty. Yet the relationship between cancer therapy and kidney disease remains underexplored. The burgeoning area of onco-nephrology suffers from lack of guidance for clinicians who encounter difficult and often complex problems in this complicated group of patients, and development of integrated guidelines is needed [30]. The 2016 American Society of Nephrology (ASN) Onco-Nephrology Curriculum may strengthen and expand understanding of this field by underscoring risk factors of CI-AKI and suggesting preventive measures be taken in patients with GFR < 60 mL/min including limiting contrast volume, using iso-osmolar contrast, prehydration with normal saline, and discontinuation of concurrent nephrotoxic agents [31].

To our understanding, this is the first study to quantify absolute risk of renal events in a robust multicenter cohort of patients undergoing CM enhanced CT with decomposed analysis of contributing factors to include CM, renal function, and cancer diagnosis. Our analysis suggests that patients who receive CM are at higher risk versus those who do not. Additionally, risk is heightened with progressively advanced stages of CKD. Further, our results substantiate multiple prior reports that cancer patients may be more uniquely susceptible to renal events undergoing CM enhanced CT versus non-cancer patients. Vulnerability of the oncologic cohort is likely multifactorial in nature and due, in part, to a high prevalence of renal insufficiency, dehydration, cachectic condition, and serial/additive renal insults induced by multiple exposures to CM, nephrotoxic medications and chemotherapeutic regimens. Results derived from our analysis may enable significant comparison of future analyses across procedures and selected high-risk populations, ultimately driving investigative research efforts and steering quality improvement endeavors.

### Strengths and limitations

Strengths of this study include the use of a comprehensive data source and use of the hospital fixed-effect specification methodology that allowed for control of time-invariant within hospital variation that is otherwise unobservable, such as physician preferences and internal protocols. The limitations of this study are those that are inherent in retrospective database analyses, which include the unit of inference (which is the visit not the patient) and potential under coding of non-billable events. The data source for this study was the Premier Healthcare Database that represents 20% of all inpatient discharges in the US; however, given its reliance on ICD-9 Codes, there is a potential risk of coding errors. A second limitation of this data source is that it does not track patients longitudinally. Thus, it was not possible to determine if events occurred after the patient was discharged. Due to the administrative nature of the database, laboratory values (sCr and GFR) were not available, we could not define CI-AKI by sCr, and rather, the outcome was defined by the ICD-9 code for CI-AKI which may underestimate the occurrence of this event. Finally, due to limitations of the dataset, we were unable to ascertain total volumes of CM administered, use of hydration strategies, or concomitant use of nephrotoxic medications or chemotherapeutic regimens.

## Conclusions

This large retrospective multicenter study decomposed the risk of acute renal events among hospitalized cancer patients having CT either with or without iodinated CM. The baseline risk for an acute renal event in patients without cancer or CKD and no CT or CM was 0.5%. When a CT procedure was performed with iodinated CM the risk increased to 1.5%. Patients with CKD having a CT with CM had an increased risk of an acute renal event from 2.5 to 8.1% depending on the stage of CKD. Among cancer patients, the overall risk increased from baseline by 0.9%. Risk increase from baseline by type of cancer ranged from 0.3 for endocrine and lung cancer to over 2% for leukemia and urinary cancer. Therefore, cancer patients having CT with iodinated CM without CKD have a risk increase of 2.4% and when CKD is present the risk ranges from 4.9 to 10.5% depending on CKD stage. In the changing healthcare landscape, with complex inpatient admissions, understanding the underlying risks of acute renal events will be an important consideration in treatment choices for oncology patients.

## Appendix

**Table 5:** Cancer Coding

ICD-9 Diagnosis Code 3 Digit Group	ICD-9 Diagnosis Code Group Description	Cancer Category
140	MALIGNANT NEOPLASM LIP	Gastrointestinal
141	MALIG NEO TONGUE	Gastrointestinal
142	MAL NEO MAJOR SALIVARY	Gastrointestinal
143	MALIGNANT NEOPLASM GUM	Gastrointestinal
144	MALIG NEO MOUTH FLOOR	Gastrointestinal
145	MALIG NEO MOUTH NEC/NOS	Gastrointestinal
146	MALIG NEO OROPHARYNX	Gastrointestinal
147	MALIG NEO NASOPHARYNX	Respiratory
148	MALIG NEOPL HYPOPHARYNX	Respiratory
149	OTH MALIG NEO OROPHARYNX	Gastrointestinal
150	MALIGNANT NEO ESOPHAGUS	Gastrointestinal
151	MALIGNANT NEO STOMACH	Gastrointestinal
152	MALIG NEO SMALL BOWEL	Gastrointestinal
153	MALIGNANT NEOPLASM COLON	Colorectal
154	MALIG NEO RECTUM/ANUS	Colorectal
155	MALIGNANT NEOPLASM LIVER	Liver
156	MAL NEO GB/EXTRAHEPATIC	Gastrointestinal
157	MALIGNANT NEO PANCREAS	Gastrointestinal
158	MALIG NEO PERITONEUM	Gastrointestinal
159	OTH MALIG NEO GI/PERITON	Gastrointestinal
160	MAL NEO NASAL CAV/SINUS	Respiratory
161	MALIGNANT NEO LARYNX	Respiratory
162	MAL NEO TRACHEA/LUNG	Lung
163	MALIGNANT NEOPL PLEURA	Lung
164	MAL NEO THYMUS/MEDIASTIN	Lung
165	OTH/ILL-DEF MAL NEO RESP	Miscellaneous
170	MAL NEO BONE/ARTIC CART	Bone
171	MAL NEO SOFT TISSUE	Miscellaneous
172	MALIGNANT MELANOMA SKIN	Skin
173	OTHER MALIG NEOPL SKIN	Skin
174	MALIG NEO FEMALE BREAST	Breast
175	MALIG NEO MALE BREAST	Breast
176	KAPOSI’S SARCOMA	Miscellaneous
179	NEOPLASM, MALIGNANT, UTERUS NEC	Gynecological
180	MALIG NEOPL CERVIX UTERI	Gynecological
181	NEOPLASM, MALIGNANT, PLACENTA	Gynecological
182	MALIG NEOPL UTERUS BODY	Gynecological
183	MAL NEO UTERINE ADNEXA	Gynecological
184	MAL NEO FEM GEN NEC/NOS	Gynecological
185	NEOPLASM, MALIGNANT, PROSTATE	Urinary
186	MALIGN NEOPL TESTIS	Urinary
187	MAL NEO MALE GENITAL NEC	Urinary
188	MALIGN NEOPL BLADDER	Urinary
189	MAL NEO URINARY NEC/NOS	Urinary
190	MALIGNANT NEOPLASM EYE	Neurological
191	MALIGNANT NEOPLASM BRAIN	Neurological
192	MAL NEO NERVE NEC/NOS	Neurological
193	NEOPLASM, MALIGNANT, THYROID GLAND	Endocrine
194	MAL NEO OTHER ENDOCRINE	Endocrine
195	MAL NEO OTH/ILL-DEF SITE	Miscellaneous
196	MALIG NEO LYMPH NODES	Hemolymph
197	SECONDRY MAL NEO GI/RESP	Gastrointestinal
198	SEC MALIG NEO OTH SITES	Miscellaneous
199	MALIGNANT NEOPLASM NOS	Miscellaneous
200	LYMPHOSARC/RETICULOSARC	Hemolymph
201	HODGKIN’S DISEASE	Hemolymph
202	OTH MAL NEO LYMPH/HISTIO	Hemolymph
203	MULTIPLE MYELOMA ET AL	Leukemia
204	LYMPHOID LEUKEMIA	Leukemia
205	MYELOID LEUKEMIA	Leukemia
206	MONOCYTIC LEUKEMIA	Leukemia
207	OTHER SPECIFIED LEUKEMIA	Leukemia
208	LEUKEMIA-UNSPECIF CELL	Leukemia
209.0×-209.3×	NEUROENDOCRINE TUMORS	Endocrine
230	CA IN SITU DIGESTIVE ORG	Gastrointestinal
231	CA IN SITU RESPIRATORY	Respiratory
232	CARCINOMA IN SITU SKIN	Skin
233	CA IN SITU BREAST/GU	Breast
234	CA IN SITU NEC/NOS	Miscellaneous
235	UNC BEHAV NEO GI/RESP	Gastrointestinal
236	UNC BEHAV NEO GU	Urinary
237	UNCER NEO ENDOCRINE/NERV	Endocrine
238	UNC BEHAV NEO NEC/NOS	Miscellaneous
239	UNSPECIFIED NEOPLASM	Miscellaneous

**Table 6:** Safety Events

Adverse Event Category	ICD-9 Diagnosis Code(s)
Acute Kidney Injury	584.9
Contrast-induced nephropathy (CIAKI)	584.9 + E947.8
Acute Kidney Injury requiring dialysis	584.9 + 39.95
Renal Failure	586.x

**Table 7:** Radiologic Imaging

Code	Description	Sub-Category	Category
ICD-9			
87.03	C.A.T. SCAN OF HEAD	CT - Diagnostic	CT
87.41	C.A.T. SCAN OF THORAX	CT - Diagnostic	CT
87.71	C.A.T. SCAN OF KIDNEY	CT - Diagnostic	CT
88.01	C.A.T. SCAN OF ABDOMEN	CT - Diagnostic	CT
88.38	OTHER C.A.T. SCAN	CT - Diagnostic	CT
CPT			
70,450	CT HEAD/BRAIN W/O DYE	CT - Diagnostic	CT
70,460	CT HEAD/BRAIN W/DYE	CT - Diagnostic	CT
70,470	CT HEAD/BRAIN W/O & W/DYE	CT - Diagnostic	CT
70,480	CT ORBIT/EAR/FOSSA W/O DYE	CT - Diagnostic	CT
70,481	CT ORBIT/EAR/FOSSA W/DYE	CT - Diagnostic	CT
70,482	CT ORBIT/EAR/FOSSA W/O&W/DYE	CT - Diagnostic	CT
70,486	CT MAXILLOFACIAL W/O DYE	CT - Diagnostic	CT
70,487	CT MAXILLOFACIAL W/DYE	CT - Diagnostic	CT
70,488	CT MAXILLOFACIAL W/O & W/DYE	CT - Diagnostic	CT
70,490	CT SOFT TISSUE NECK W/O DYE	CT - Diagnostic	CT
70,491	CT SOFT TISSUE NECK W/DYE	CT - Diagnostic	CT
70,492	CT SFT TSUE NCK W/O & W/DYE	CT - Diagnostic	CT
70,496	CT ANGIOGRAPHY HEAD	CT Angiography - Diagnostic	CTA
70,498	CT ANGIOGRAPHY NECK	CT Angiography - Diagnostic	CTA
71,250	CT THORAX W/O DYE	CT - Diagnostic	CT
71,260	CT THORAX W/DYE	CT - Diagnostic	CT
71,270	CT THORAX W/O & W/DYE	CT - Diagnostic	CT
71,275	CT ANGIOGRAPHY CHEST	CT Angiography - Diagnostic	CTA
72,125	CT NECK SPINE W/O DYE	CT - Diagnostic	CT
72,126	CT NECK SPINE W/DYE	CT - Diagnostic	CT
72,127	CT NECK SPINE W/O & W/DYE	CT - Diagnostic	CT
72,128	CT CHEST SPINE W/O DYE	CT - Diagnostic	CT
72,129	CT CHEST SPINE W/DYE	CT - Diagnostic	CT
72,130	CT CHEST SPINE W/O & W/DYE	CT - Diagnostic	CT
72,131	CT LUMBAR SPINE W/O DYE	CT - Diagnostic	CT
72,132	CT LUMBAR SPINE W/DYE	CT - Diagnostic	CT
72,133	CT LUMBAR SPINE W/O & W/DYE	CT - Diagnostic	CT
72,191	CT ANGIOGRAPH PELV W/O&W/DYE	CT Angiography - Diagnostic	CTA
72,192	CT PELVIS W/O DYE	CT - Diagnostic	CT
72,193	CT PELVIS W/DYE	CT - Diagnostic	CT
72,194	CT PELVIS W/O & W/DYE	CT - Diagnostic	CT
73,200	CT UPPER EXTREMITY W/O DYE	CT - Diagnostic	CT
73,201	CT UPPER EXTREMITY W/DYE	CT - Diagnostic	CT
73,202	CT UPPR EXTREMITY W/O&W/DYE	CT - Diagnostic	CT
73,206	CT ANGIO UPR EXTRM W/O&W/DYE	CT Angiography - Diagnostic	CTA
73,700	CT LOWER EXTREMITY W/O DYE	CT - Diagnostic	CT
73,701	CT LOWER EXTREMITY W/DYE	CT - Diagnostic	CT
73,702	CT LWR EXTREMITY W/O&W/DYE	CT - Diagnostic	CT
73,706	CT ANGIO LWR EXTR W/O&W/DYE	CT Angiography - Diagnostic	CTA
74,150	CT ABDOMEN W/O DYE	CT - Diagnostic	CT
74,160	CT ABDOMEN W/DYE	CT - Diagnostic	CT
74,170	CT ABDOMEN W/O & W/DYE	CT - Diagnostic	CT
74,175	CT ANGIO ABDOM W/O & W/DYE	CT Angiography - Diagnostic	CTA
74,261	CT COLONOGRAPHY DX	CT - Diagnostic	CT
74,262	CT COLONOGRAPHY DX W/DYE	CT - Diagnostic	CT
74,263	CT COLONOGRAPHY SCREENING	CT - Diagnostic	CT
75,571	CT HRT W/O DYE W/CA TEST	CT - Diagnostic	CT
75,572	CT HRT W/3D IMAGE	CT - Diagnostic	CT
75,573	CT HRT W/3D IMAGE CONGEN	CT - Diagnostic	CT
75,574	CT ANGIO HRT W/3D IMAGE	CT Angiography - Diagnostic	CTA
75,635	CT ANGIO ABDOMINAL ARTERIES	CT Angiography - Diagnostic	CTA
76,380	CAT SCAN FOLLOW-UP STUDY	CT - Diagnostic	CT
76,497	CT PROCEDURE	CT - Diagnostic	CT
77,011	CT SCAN FOR LOCALIZATION	CT - Guidance	CT
77,012	CT SCAN FOR NEEDLE BIOPSY	CT - Guidance	CT
77,013	CT GUIDE FOR TISSUE ABLATION	CT - Guidance	CT
77,014	CT SCAN FOR THERAPY GUIDE	CT - Guidance	CT
77,078	CT BONE DENSITY AXIAL	CT - Diagnostic	CT
77,079	CT BONE DENSITY, PERIPHERAL	CT - Diagnostic	CT
0042 T	CT PERFUSION W/CONTRAST, CBF	CT - Diagnostic	CT & CTA
S8092	ELECTRON BEAM COMPUTED TOMOG	CT - Diagnostic	CT

**Table 8:** Chronic Kidney Disease

Chronic Kidney Disease Stage	ICD-9 Diagnosis Code(s)
Chronic Kidney Disease Stage 1	585.1
Chronic Kidney Disease Stage 2	585.2
Chronic Kidney Disease Stage 3	585.3
Chronic Kidney Disease Stage 4	585.4
Chronic Kidney Disease Stage 5	585.5
Chronic Kidney Disease, unspecified	585.9

**Table 9:** Elixhauser Comorbidity Index*

Comorbidity	Codes
Congestive Heart Failure	398.91, 402.01, 402.11, 402.91, 404.01, 404.03, 404.11, 404.13, 404.91, 404.93, 425.4–425.9, 428.x
Cardiac Arrhythmia	426.0, 426.13, 426.7, 426.9, 426.10, 426.12, 427.0–427.4, 427.6–427.9, 785.0, 996.01, 996.04, V45.0, V53.3
Valvular Disease	093.2, 394.x–397.x, 424.x, 746.3–746.6, V42.2, V43.3
Pulmonary Circulation Disorders	415.0, 415.1, 416.x, 417.0, 417.8, 417.9
Peripheral Vascular Disorders	093.0, 437.3, 440.x, 441.x, 093.0, 437.3, 440.x, 441.x, 443.1–443.9, 447.1, 557.1, 557.9, V43.4
Hypertension (Uncomplicated)	401.x
Hypertension (Complicated)	402.x–405.x
Paralysis	334.1, 342.x, 343.x, 344.0–344.6, 344.9
Other Neurological Disorders	331.9, 332.0, 332.1, 333.4, 333.5, 333.92, 334.x–335.x, 336.2, 340.x, 341.x, 345.x, 348.1, 348.3, 780.3, 784.3
Chronic Pulmonary Disease	416.8, 416.9, 490.x − 505.x, 506.4, 508.1, 508.8
Diabetes (Uncomplicated)	250.0–250.3
Diabetes (Complicated)	250.4–250.9
Hypothyroidism	240.9, 243.x, 244.x, 246.1, 246.8
Renal Failure	403.01, 403.11, 403.91, 404.02, 404.03, 404.12, 404.13, 404.92, 404.93, 585.x, 586.x, except 585.6, 588.0, V42.0, V45.1, V56.x
End-stage renal disease	585.6
Liver Disease	070.22, 070.23, 070.32, 070.33, 070.44, 070.54, 070.6, 070.9, 456.0–456.2, 570.x, 571.x, 572.2–572.8, 573.3, 573.4, 573.8, 573.9, V42.7
Peptic Ulcer Disease (excluding bleeding)	531.7, 531.9, 532.7, 532.9, 533.7, 533.9, 534.7, 534.9
AIDS/HIV	042.x–044.x
Lymphoma	200.x–202.x, 203.0, 238.6
Metastatic Cancer	196.x–199.x
Solid Tumor without Metastasis	140.x–172.x, 174.x–195.x
Rheumatoid Arthritis Collagen	446.x, 701.0, 710.0–710.4, 710.8, 710.9, 711.2, 714.x, 719.3, 720.x, 725.x, 728.5, 728.89, 729.30
Coagulopathy	286.x, 287.1, 287.3–287.5
Obesity	278.0
Weight Loss	260.x–263.x, 783.2, 799.4
Fluid and Electrolyte Disorders	253.6, 276.x
Blood Loss Anemia	280.0
Deficiency Anemia	280.1–280.9, 281.x
Alcohol Abuse	265.2, 291.1–291.3, 291.5–291.9, 303.0, 303.9, 305.0, 357.5, 425.5, 535.3, 571.0–571.3, 980.x, V11.3
Drug Abuse	292.x, 304.x, 305.2–305.9, V65.42
Psychoses	293.8, 295.x, 296.04, 296.14, 296.44, 296.54, 297.x, 298.x
Depression	296.2, 296.3, 296.5, 300.4, 309.x, 311

*The ECI Score includes 31 categories (Table 9 in Appendix) of comorbidities, which are associated with mortality. Each category counts as 1 point for a potential ECI score range of 0–31. These comorbidities were identified using diagnosis codes that appear during the visit

## Abbreviations

AE: Adverse event(s)
AKI: Acute kidney injury
ASN: American Society of Nephrology
CI-AKI: Contrast-induced acute kidney injury
CIN: Contrast-induced nephropathy
CKD: Chronic kidney disease
CM: Contrast material
CT: Computed tomography
ECI: Elixhauser comorbidity index
ESRD: End stage renal disease
ICD-9: International classification of diseases, ninth revision
NHDS: National Hospital Discharge Survey
PC-AKI: Post-contrast acute kidney injury
SCr: Serum creatinine
US: United States
